# En Bloc Resection of Bladder Tumors (ERBT) using different lasers – Hybrid and Holmium Laser

**DOI:** 10.1590/S1677-5538.IBJU.2023.0231

**Published:** 2024-02-07

**Authors:** Alexandre Iscaife, Moises Rodríguez Socarras, Luis Llanes González, Juan Gómez Rivas, Maykon William Aparecido Pires Pereira, Katia Ramos Moreira Leite, Willian Carlos Nahas, Fernando Gomez Sancha

**Affiliations:** 1 Universidade de São Paulo Instituto do Câncer do Estado de Sao Paulo Sao Paulo Brasil Instituto do Câncer do Estado de Sao Paulo (ICESP) – Universidade de São Paulo, Sao Paulo, Brasil;; 2 Clínica Cemtro Instituto de Cirugia Urologica Avanzada Madrid Spain Instituto de Cirugia Urologica Avanzada (ICUA) - Clínica Cemtro, Madrid-Spain

## Abstract

**Introduction:**

The En-bloc Resection of Bladder Tumors (ERBT) is a method that offers more benefits compared to the traditional Transurethral Resection of Bladder Tumor (TURBT) (1, 2). Recent studies have shown that ERBT offers better pathological analysis and oncological outcomes (3-6). Thulium and holmium are the most frequently used lasers for this procedure, with the hybrid laser being a new addition that combines thulium and diode to improve hemostatic properties (5, 7-9).

**Objective:**

This report aims to discuss the use of two types of lasers, hybrid and holmium, for ERBT.

**Material and Methods:**

Two case studies were conducted. The first case featured a 68-year-old male with two tumors measuring 1.5cm and 2cm. The hybrid laser was used for the procedure. The second case involved a 70-year-old female with a 5cm tumor on the posterior bladder wall, and holmium laser was used with morcellation of the tumor. The quality of histopathological analysis was evaluated. The perioperative data and the entire procedure of the two cases were documented in a step-by-step video.

**Results:**

Both lasers demonstrated excellent results without technical difficulties. There was no bleeding, and both patients were discharged with one day of hospitalization. The detrusor muscle was present without artifacts, and the morcellation did not affect the analysis. The first case showed a pT1G3, and the second case showed a pT2 urothelial carcinoma. The hybrid laser exhibited superior hemostatic capacity compared to the holmium laser.

**Conclusion:**

ERBT can use hybrid or holmium lasers without affecting histopathological analysis, even with morcellation.

**Available at:** http://www.intbrazjurol.com.br/video-section/20230231_Iscaife_et_al

**Int Braz J Urol. 2023; 49 (Video #13): 783-4**

## References

[1] Baird B, Bilgili A, Anderson A, Carames G, Pathak RA, Ball CT (2023). Oncological outcomes of visibly complete transurethral resection prior to neoadjuvant chemotherapy for bladder cancer. Int Braz J Urol.

[2] Carneiro A. (2022). The management of muscle-invasive bladder Cancer is still a significant challenge in the clinical practice. Int Braz J Urol.

[3] Hashem A, Mosbah A, El-Tabey NA, Laymon M, Ibrahiem EH, Elhamid MA (2021). Holmium Laser En-bloc Resection Versus Conventional Transurethral Resection of Bladder Tumors for Treatment of Non-muscle-invasive Bladder Cancer: A Randomized Clinical Trial. Eur Urol Focus.

[4] Li C, Gao L, Zhang J, Yang X, Liu C. (2020). The effect of holmium laser resection versus standard transurethral resection on non-muscle-invasive bladder cancer: a systematic review and meta-analysis. Lasers Med Sci.

[5] Kramer MW, Wolters M, Cash H, Jutzi S, Imkamp F, Kuczyk MA (2015). Current evidence of transurethral Ho:YAG and Tm:YAG treatment of bladder cancer: update 2014. World J Urol.

[6] Teoh JY, MacLennan S, Chan VW, Miki J, Lee HY, Chiong E (2020). An International Collaborative Consensus Statement on En Bloc Resection of Bladder Tumour Incorporating Two Systematic Reviews, a Two-round Delphi Survey, and a Consensus Meeting. Eur Urol.

[7] Zhang W, Zhou B, Deng J, Han G, Ni W, Nie Q. (2023). Retrospective analysis of 1470-/980-nm dual-wavelength laser en bloc resection versus transurethral resection of bladder tumor for primary non-muscle-invasive bladder cancer. Lasers Med Sci.

[8] Fu J, Fu F, Wang Y. (2021). 1470 nm/980 nm dual-wavelength laser is safe and efficient for the en-bloc resection of non-muscle invasive bladder cancer: A propensity score-matched analysis. J Int Med Res.

[9] Badawy A, Sultan SM, Marzouk A, El-Sherif E. (2023). Thulium laser en bloc resection versus conventional transurethral resection of urinary bladder tumor: A comparative prospective study. Urol Ann.

